# Enhancing EPA Content in an Arctic Diatom: A Factorial Design Study to Evaluate Interactive Effects of Growth Factors

**DOI:** 10.3389/fpls.2018.00491

**Published:** 2018-04-17

**Authors:** Pia Steinrücken, Svein A. Mjøs, Siv K. Prestegard, Svein R. Erga

**Affiliations:** ^1^Department of Biological Sciences, University of Bergen, Bergen, Norway; ^2^Department of Chemistry, University of Bergen, Bergen, Norway; ^3^Applied Biotechnology, Uni Research Environment, Bergen, Norway

**Keywords:** eicosapentaenoic acid (EPA), arctic diatom, factorial design, salinity, growth phase, interactive effects, microalgal biotechnology

## Abstract

Microalgae with a high content of the omega-3 polyunsaturated fatty acids (PUFAs), eicosapentaenoic acid (EPA), and docosahexaenoic acid (DHA) are of great demand for microalgae-based technologies. An Arctic strain of the diatom *Attheya septentrionalis* was shown in previous experiments to increase its EPA content from 3.0 to 4.6% of dry weight (DW) in the nutrient-replete exponential phase and nutrient-depleted stationary phase, respectively. In the present study, a factorial-design experiment was used, to investigate this effect in more detail and in combination with varying salinities and irradiances. A mathematical model revealed that both growth phase and salinity, alone and in combination, influenced the EPA content significantly. Maximum EPA values of 7.1% DW were obtained at a salinity of 22 and after 5 days in stationary phase, and might be related to a decreased silica content, an accumulation of storage lipids containing EPA, or both. However, growth rates were lower for low salinity (0.54 and 0.57 d^−1^) than high salinity (0.77 and 0.98 d^−1^) cultures.

## Introduction

The omega-3 polyunsaturated fatty acids (PUFAs), eicosapentaenoic acid (EPA), and docosahexaenoic acid (DHA) are known to contribute significantly to human health (Martins et al., 2013). The current major source for the two fatty acids (FA) is fish oil from marine wild fish. The fish obtain and accumulate these PUFAs themselves predominantly via the marine food chain from EPA- and DHA-synthesizing microalgae (Spolaore et al., 2006). As EPA and DHA are also essential for farmed fish (Khozin-Goldberg et al., 2016), fish oil is an important additive in aquaculture feed. The increasing demand for EPA and DHA, particularly from the growing aquaculture industries, but also increasingly from the health and food sectors, necessitates other sustainable production sources. Many marine microalgae naturally produce EPA and DHA, and are therefore considered a promising alternative (Patil et al., 2005; Chauton et al., 2015). Although there has been intensive research in this field, the costs associated with microalgae large-scale cultivation and processing for FAs are still greater than in fish oil production. Improvements at the different parts of the production chain are therefore essential in order to reduce production costs. The selection of suitable species that are superior to existing cultures in terms of growth and EPA and DHA content, and the optimization of cultivation conditions are important contributions to the ongoing improvement of microalgae-based technologies (Adarme-Vega et al., 2012).

In previous research, we searched for new and fast growing microalgal strains from North Atlantic habitats with high growth rates and high EPA and DHA contents (Steinrücken et al., 2017). A strain of the diatom *Attheya septentrionalis*, isolated from Arctic Waters, demonstrated rapid growth at temperatures of 10°C (0.7 d^−1^) and a high EPA content, which increased from 3.0 to 4.6% of the dry weight (DW) from exponential phase to Day 3 of the stationary phase. This EPA content under nutrient-depleted conditions was higher than typically found in industrially applied microalgae and this strain was therefore suggested to be a potential candidate for future large-scale cultivation and EPA production. A high growth rate and EPA content in stationary phase in *A. septentrionalis* were also found by Knuckey et al. (2002) who suggested this diatom to be a promising feed source for oyster hatcheries. However, only few studies on *Attheya* species exist (Aizdaicher and Markina, 2011; Stonik et al., 2017), and more explicit investigations are needed in order to assess the potential of this diatom for microalgae-based technologies.

It is well-known that microalgae modify their biochemical composition and FA content in response to environmental factors, including nutrient availability, irradiance, temperature, and salinity (Dunstan et al., 1993; Renaud and Parry, 1994; Tatsuzawa and Takizawa, 1995; Xu and Beardall, 1997; Van Wagenen et al., 2012; Boelen et al., 2013; Cepák et al., 2013). To investigate the impact of the different factors, traditional methods vary one condition at a time, while keeping all other factors constant. However, FA composition and content in microalgae are dependent on synergistic and antagonistic interactions of cultivation conditions. Factorial designs are based on a multivariate approach, in which the variation in different factors are tested simultaneously (Duarte et al., 2001). These yield a predictive model which provides information on the magnitude of the effects of both individual factors and combinations of factors, and on their statistical significance (Chen et al., 2012).

In this study, we aimed to elucidate additional information on the dynamics of the EPA content and relative FA composition in *A. septentrionalis*, and to determine the experimental conditions that might lead to a further increase in the EPA content. A factorial-design experiment was used to investigate the impact of nutrient starvation in greater detail, together with the effects of salinity and irradiance, and their respective interactions. Both salinity and irradiance are known to affect the FA composition in microalgae (Xu and Beardall, 1997; Lu et al., 2001; Chen et al., 2008), and are highly variable in Arctic environments due to melting and freezing sea ice, and strong variations in photoperiod. Hence, microalgae from these environments are expected to be promising in this context, and possess the necessary adaptations to cope with these changing conditions.

## Methods

### Stock cultures and inoculum

*Attheya septentrionalis* is a single celled diatom with four long setae, and is broadly distributed in Arctic and Temperate Waters (Rampen et al., 2009; Stonik et al., 2017). The strain used in this experiment was isolated from Arctic Waters north-west of Spitzbergen (N 79° 25.14′ E 08° 18.84′) in May 2014 (3.5°C water temperature, salinity of 35, and 24 h daylight). For strain characterization, a region of the 28S ribosomal RNA (rRNA) gene was sequenced and compared with previously published sequences from diatoms at GenBank (Steinrücken et al., 2017). Partial sequence of the 28S rRNA gene have been deposited in GenBank (http://www.ncbi.nlm.nih.gov/) with accession number MH020639. Stock cultures were maintained in 50 mL Erlenmeyer flasks at 10°C and 50 μmol photons m^−2^ s^−1^, in nutrient-enriched (Walne, 1970) 80% seawater (SW) by monthly dilution. Eighty percent SW was obtained by dilution of filtered and autoclaved fjord-SW (from 90 m depth, salinity of 35) with distilled water (80:20, v:v), giving a salinity of 29. For the inoculum, biomass was harvested from exponential phase stock cultures by centrifugation (2,264 × g, 5 min), washed twice with fresh medium and re-inoculated into 10 mL fresh medium.

### Experimental design

Factorial design was used to investigate the effects of salinity, irradiance, and growth phase and their interactions on the EPA and total fatty acid (TFA) content, and the FA composition in the diatom *A. septentrionalis*, by growth of batch cultures at different conditions. The effects of salinity and irradiance were assessed at two levels (22 and 35, and 50 and 200 μmol photons m^−2^ s^−1^, respectively), and the growth phase at three levels; exponential phase (e), Day 3 of stationary phase (first stationary phase, s1), and Day 5 of stationary phase (second stationary phase, s2), resulting in 12 treatment groups. Salinity and irradiance levels were selected as being representative of those occurring under natural conditions in the Arctic and additionally, so as to induce different responses but not to impair the cultures. Before the start of the batch experiment, pre-cultures (one biological replicate for each condition) were grown semi-continuously for 14 d at either low or high salinity and low or high irradiance (LSLI, HSLI, LSHI, and HSHI), to acclimate the cultures to their respective conditions. Two sterile and nutrient enriched media (Walne, 1970) were prepared with aged SW (salinity of 35) and respective dilutions with distilled water (salinity of 22). Pre-cultures were prepared in glass tubes (300 mL, 3.5 cm inner diameter), by adding 1 mL inoculum to 60 mL fresh medium of the respective salinity, and placed into temperature-controlled water tanks (10°C). Continuous illumination with 50 or 200 μmol photons m^−2^ s^−1^ (measured with 4π quantum scalar irradiance sensor [QSL-100, Biospherical Instruments, San Diego, CA, USA], inside the empty glass cylinder) was provided by banks of six white fluorescent tubes (Philips MASTER, TL-D 90 Graphica, 58W/95) in the back of the water tanks, running perpendicular to the glass tubes. To ensure adequate mixing and carbon supply, 0.2 μm-filtered and 1% CO_2_-enriched air was bubbled through glass capillaries into the bottom of each glass tube. Pre-cultures were kept in exponential phase by maintaining an optical density (OD_750_) between 0.15 and 0.50, by the addition of fresh medium, successively added until volumes reached 260 mL. Thereafter, half of the culture volume was replaced with fresh medium daily or every other day. After the 2-week acclimation period, the batch experiment was started. Biomass from each pre-culture was distributed into two sterile glass tubes to obtain two biological replicates, and each diluted with fresh medium to yield a starting OD and volume of ~0.15 and 260 mL, respectively. Cultures were then grown until Day 5 of the stationary phase, and all cultures were sampled daily for OD and maximum quantum yield (QY), and on Day 1 in exponential phase (e) and Days 3 and 5 in stationary phase (s1 and s2, respectively) for DW and FA analysis. Additionally, cultures were sampled for nutrient analysis of the media at the start of the experiment, and on the DW and FA sampling days.

### Analytical procedures

Relative growth rates between repeated dilutions during pre-cultivation were calculated according to the changes in attenuation with Equation (1). *N*_*x*0_ and *N*_*x*_ are OD_750_ after dilution (*t*_*x*0_) and before the subsequent dilution (*t*_*x*_), respectively.

(1) $$\mu \;\left(d^{-1}\right)=\frac{\text{ ln}\left(N_{x}\right)-\ln(N_{x0})}{\left(t_{x}-t_{x0}\right)}$$

Optical density measurements were performed using a spectrophotometer (UV1800, Shimadzu Corporation, Kyoto, Japan) at 750 nm and if required, samples were diluted to give an attenuation between 0.2 and 0.8. The QY was measured with AquaPen (AquaPen-C, AP-C 100, Photon System Instruments, Brno, Czech Republic) after initial dark incubation between 10 and 60 min. DWs (expressed as weight of the dried biomass [g] per volume [L]) were determined in triplicates as described by Zhu and Lee (1997), with 0.5 M ammonium formate as a washing buffer.

For FA analysis, triplicate 10 mL microalgal cultures were harvested by centrifugation (6 min at 2,264 × g) into glass tubes (PYREX), the supernatant discarded, and the pellet covered in nitrogen atmosphere and stored at −20°C until analysis. FAs were extracted and derivatized to fatty acid methyl esters (FAME) by direct esterification (Meier et al., 2006). The pellet was dried in the 10 mL tube by evaporating the remaining water under a nitrogen stream, and 18 or 34 μg internal standard (23:0 FAME dissolved in isooctane) was added to exponential or stationary phase samples, respectively. The solvent was evaporated under nitrogen stream and 0.5 mL methylation reagent (2M HCl in methanol) was added. Samples were covered with nitrogen gas, sealed and incubated at 90°C for 2 h. After cooling to room temperature, half of the methylation reagent was evaporated, 0.5 mL water was added, and the samples were extracted twice with 1 mL isooctane. The combined extracts of stationary phase samples were diluted with isooctane (1:1, v:v) to yield a final internal standard concentration of approximately 18 μg mL^−1^ (Steinrücken et al., 2017). FAMEs were analyzed by GC (7890 gas chromatograph, Agilent, Santa Clara, CA, USA) equipped with an autosampler, split-splitless injector, flame ionization detector (FID), and a 60 m BPX70 capillary column (SGE, Ringwood, Australia) with internal diameter of 0.25 mm and film thickness of 0.25 μm. One microliters of sample volumes were injected splitless at 60°C. This temperature was maintained for 3 min before raised by 40°C min^−1^ to 150°C and by 1.5°C min^−1^ to 230°C. Helium, with an estimated average velocity of 30 cm s^−1^ was used as carrier gas in constant flow mode. Injector and detector temperatures were 250 and 300°C, respectively (Prestegard et al., 2015). The FAMEs were identified by analysis on gas chromatography coupled to mass spectrometry (GC-MS) as described in Wasta and Mjøs (2013), and by using libraries of mass spectra and retention indices available at www.chrombox.org/data.

For nutrient measurements of the media, 20 mL GF/F filtrates were collected in white plastic vials, 100 μl chloroform added and stored at 4°C before analysis. Dissolved inorganic nitrate, nitrite, orthophosphate, and silicate were analyzed at the Institute of Marine Research, Bergen, which offers accredited and standardized service for nutrient analyses, using colorimetric absorption measurements on an Alpkem-Lab analyzer (Alpkem Corporation, Oregon USA) according to Parsons et al. (1992).

### Regression models

The model for growth rate as a function of salinity, irradiance and their interactions, and models for TFA and EPA content as functions of salinity, irradiance, growth phase, and their interactions were calculated by multiple least squares regression. The models reported in the paper are based on the coded factor levels, where the low values are assigned −1 and the high values are assigned +1. For the growth phase, there are three levels; exponential phase, and first and second stationary phase. Exponential and second stationary phase were assigned the levels −1 and +1, respectively. The level for first stationary phase was set to 0.74. This level was found by iteratively testing values from −1 to 1 with increments of 0.01, and selecting the value that minimized the sum of squared residuals of the model. Models and model statistics were calculated by the fitlm function in the Statistics and Machine Learning Toolbox running under Matlab R2017a (Mathworks, Natick, MA, USA).

### Statistics

The batch experiment was performed with two individual cultures (biological replicates) for each treatment, which is sufficient for solid statistics when using regression analysis and factorial design. One measurement replicate was taken for OD and QY measurements, whereas *FA* and *DW* were analyzed in triplicates for each biological replicate. The *FA* content and the *DW* were analyzed from individual subsamples, and the standard deviation (*SD*) for FA content relative to the *DW* was calculated using Equation (2).

(2) $$SD_{FA/DW}=\frac{\sqrt{\%SD_{FA}{}^{2}+\%SD_{{}_{DW}}{}^{2}}}{100}\times \frac{FA}{DW}$$

Euclidean dendrograms and Principal Component Analysis (PCA) of treatment groups and their FA composition were calculated using Sirius 10.0 (Pattern Recognition Systems AS, Bergen, Norway) and edited in GraphPad Prism 6.

## Results

### Pre-cultivation—growth rates

**Figure 1:**
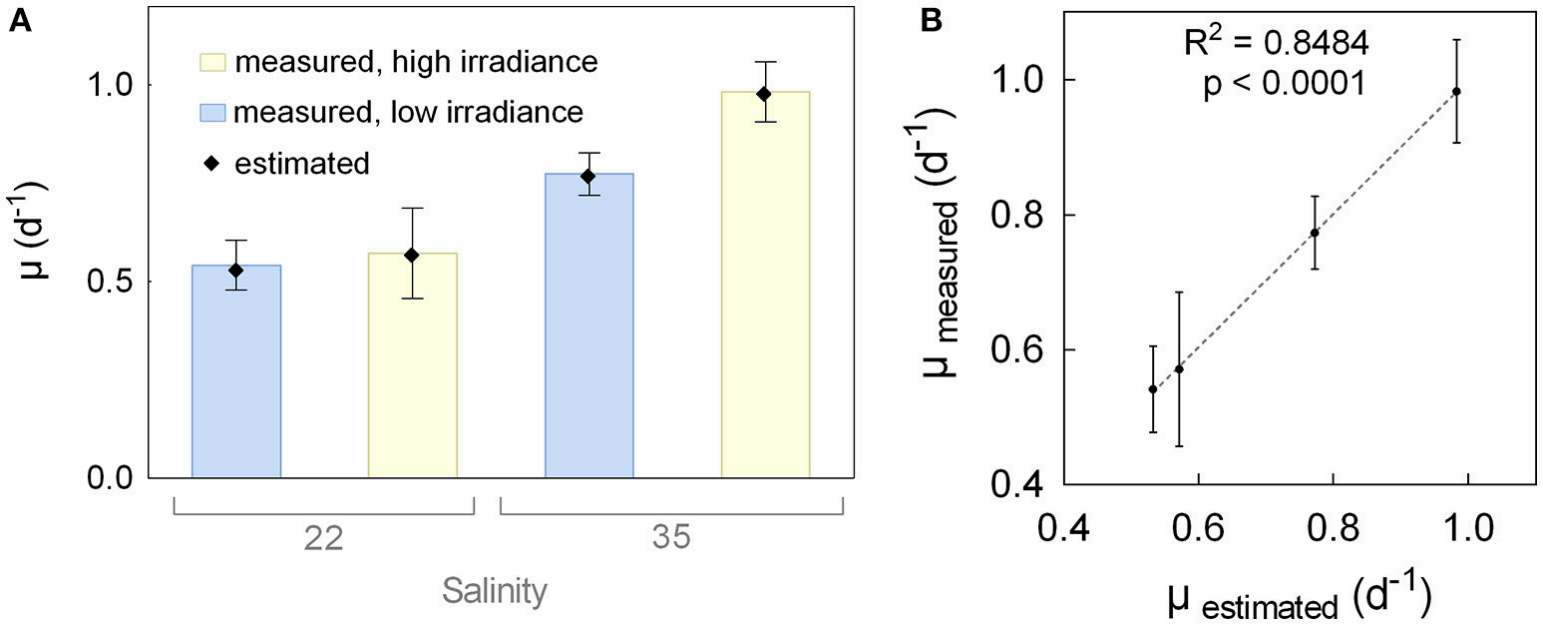
Growth rates (μ) during pre-cultivation. **(A)** Average growth rates of repeated dilutions (bars, *n* = 7) and estimated growth rates by the model (dots) of the diatom *Attheya septentrionalis* grown at four different cultivation conditions (combinations of low or high salinity [22 and 35] and irradiance [50 and 200 μmol photons m^−2^ s^−1^]. **(B)** Average and standard deviation (*n* = 7) of measured growth rates, plotted against the estimated growth rates by the model, with linear regression.

Pre-cultures were grown for 2 weeks to allow for cells to acclimate to the respective salinities and irradiances. After three dilutions, growth rates for each condition became more constant although they still varied slightly between the repeated dilutions. Only growth rates from the final seven dilutions prior to the batch experiment were used for analyses (Table S1). Average values, together with the estimates provided by the mathematical model, are shown in Figure 1A. Lower growth rates (0.54 d^−1^ for low irradiance [LI] and 0.57 d^−1^ for high irradiance [HI]) were observed for low salinity (LS) cultures, compared to high salinity (HS) cultures (0.77 d^−1^ for LI and 0.98 d^−1^ for HI), together with increased values for HI cultures. High irradiance had a stronger positive effect on the growth rate at HS. The mathematical model representing the growth rate as a function of salinity (*X1*), irradiance (*X2*), and their combination (*X1X2*) in the experimental setup is expressed by Equation (3). Positive coefficients in the equations indicate that increasing values increase the growth rate and bold numbers indicate a high statistical significance (black: *p* < 0.05 and red: *p* < 0.01). According to the model, salinity had the strongest positive influence on the growth rate, with high significance (0.163, *p*-value 1.4*10^−09^), while irradiance (0.062, *p* = 0.001) and the combination of salinity and irradiance (0.043, *p* = 0.016) had lower, but still significant, impacts. A strong and significant correlation (*R*^2^ = 0.8484) between the estimated values of the model and the measured values indicates a good fit between the model and the experimental data (Figure 1B). Details on the measured and estimated growth rates can be found in the Supplementary Material (Table S1).

(3) $$\mu \;{\left(d^{-1}\right)}_{est}=0.715+\;0.163*X1+\;0.062*X2+0.043*X1X2$$

### Batch experiment—growth and maximum quantum yield (QY)

**Figure 2:**
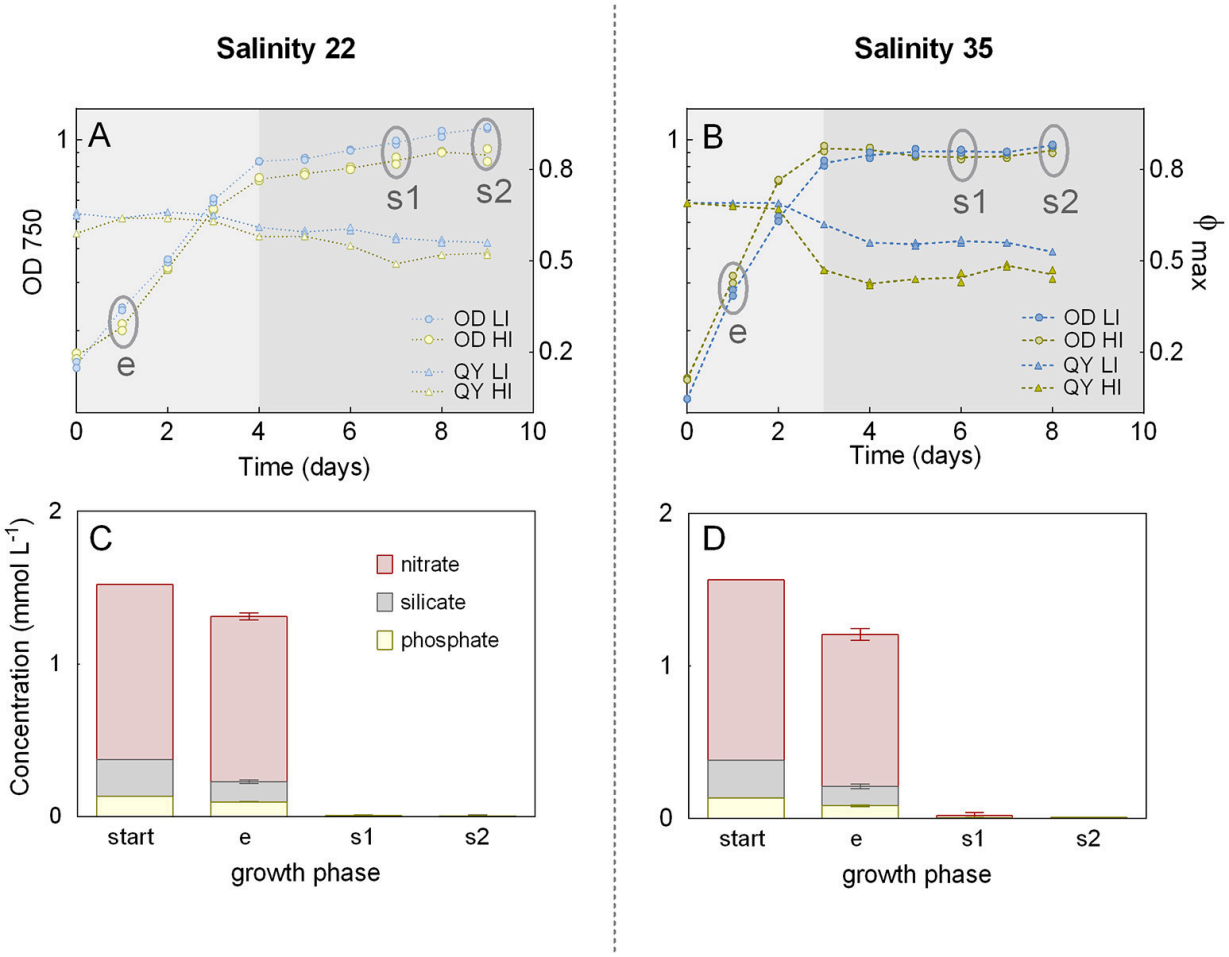
Growth during batch experiment. Batch cultures of the diatom *Attheya septentrionalis* grown at four conditions (combinations of low and high salinity [22 and 35] and irradiance [50 and 200 μmol photons m^−2^ s^−1^] with two biological replicates for each condition). **(A,B)** Optical density (OD_750_) based growth curves and maximum quantum yields (QY, Φmax) for low and high salinities, respectively. Circles indicate sampling time points (e, exponential phase; s1, first stationary phase; s2, second stationary phase). LI, low irradiance; HI, high irradiance. **(C,D)** Superimposed nutrient concentration in the media at the start of the experiment and during exponential (e) and stationary (s1 and s2) sampling time points for low and high salinities, respectively. Values are the averages and standard deviations from samples of the respective salinity.

Growth curves and QY during the batch experiment were very similar for the biological replicates, but there were differences between the cultivation conditions, especially between low and high salinities (Figures 2A,B). Cultures grown at LS, revealed a declined slope of the growth curves and reached stationary phase 1 day later (Day 4) than cultures grown at HS (Day 3). During exponential growth, the QY was slightly lower for the LS cultures (replicate averages of 0.64 at Day 1, both for low and high irradiances) than for the HS cultures (replicate averages of 0.69 and 0.68 at Day 1, for low and high irradiance, respectively). During stationary phase, the QY decreased in all cultures. In HS cultures, QY dropped by 10% (LI) and 30% (HI) after entering stationary phase, and by 23 and 32%, respectively, at the end of cultivation period. A lower reduction was observed for the LS cultures, where QY was reduced by 6 and 8% (low and high irradiance, respectively) after entering stationary phase, and by 14 and 17% at the end of cultivation.

The nitrate, silicate, and phosphate concentrations of the media decreased from the start of the experiment to the first day of exponential phase by 14, 39, and 27% for low salinity cultures and by 23, 45, and 38% for high salinity cultures, respectively (Figures 2C,D). At the first stationary phase (Day 3), all nutrients had been consumed in all cultures (nitrate 99%, silicate 99%, and phosphate 97%).

### Batch experiment—dry weights (DW), total fatty acids (TFA), and EPA

**Figure 3:**
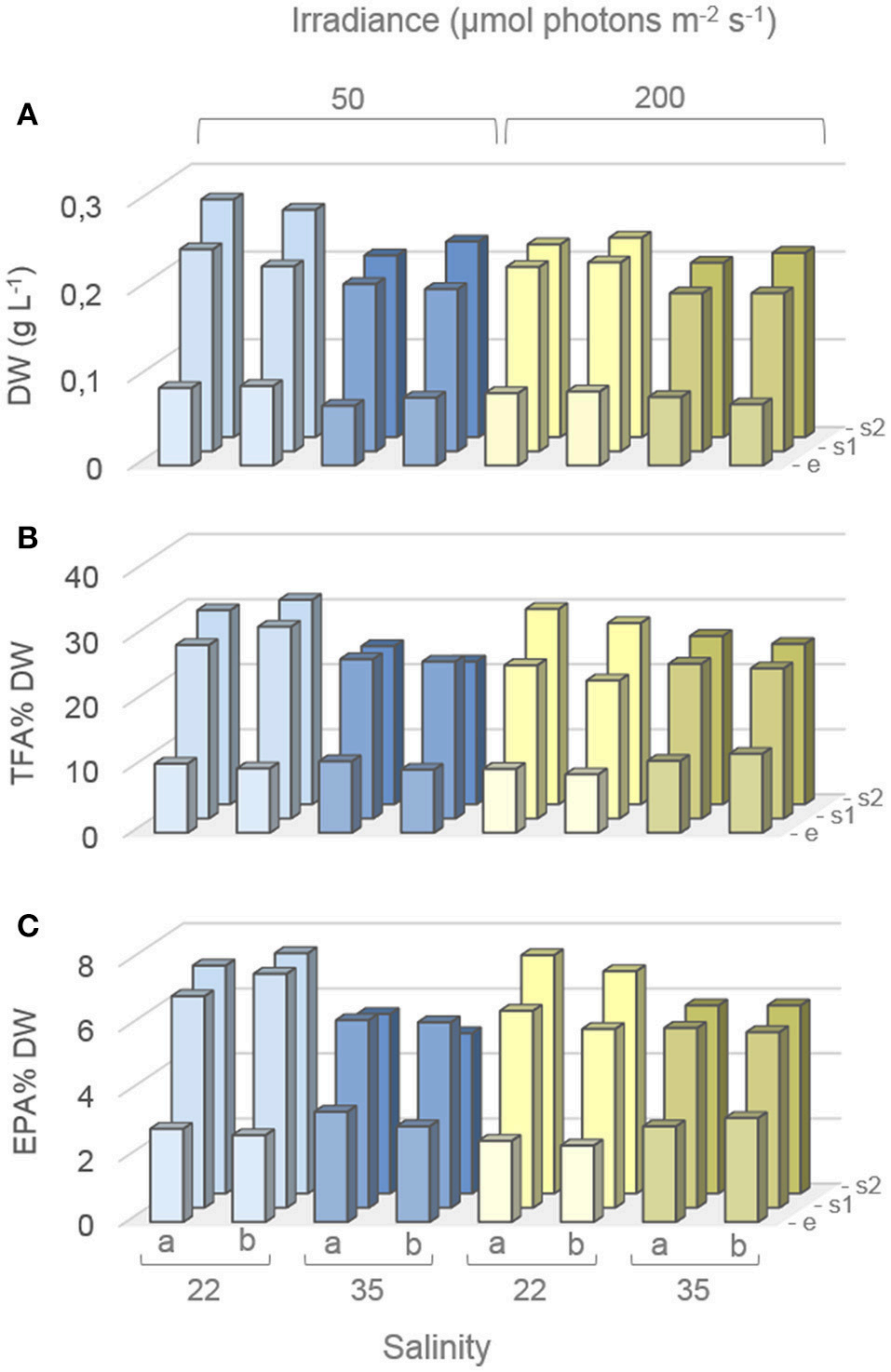
Dry weights (DW) and fatty acid content during factorial-design batch experiment. Average DW **(A)**, and total fatty acid (TFA) and EPA contents (**B,C** respectively) of *Attheya septentrionalis* cultures at 12 different treatments (two biological replicates per treatment, a and b). Values are average of three measurement replicates. Detailed values with standard deviations can be found in Table S2. e, exponential phase; s1, first stationary phase (Day3); s2, second stationary phase (Day 5).

DW together with TFA and EPA contents (% DW) for the 12 different treatment groups, each with two biological replicates are shown in Figure 3. The DW (average of the biological replicates) increased from the exponential phase to the first stationary phase by 0.13, 0.12, 0.13, and 0.11 g L^−1^ and further by 0.04, 0.03, 0.01, 0.02 g L^−1^ to the second stationary phase, giving a total increase of 0.17, 0.15, 0.14, and 0.13, g L^−1^ for low salinity-low irradiance (LSLI), high salinity-low irradiance (HSLI), low salinity-high irradiance (LSHI), and high salinity-high irradiance (HSHI) conditions, respectively (Figure 3A).

The TFA and EPA contents (Figures 3B,C respectively) increased from the exponential to the stationary phase at all growth conditions, but to different extents. In exponential phase, EPA contents (average of replicates) were 2.8, 3.2, 2.4, and 3.1% DW for LSLI, HSLI, LSHI, and HSHI conditions, respectively, and increased to 6.8, 5.7, 5.8, and 5.5% DW, respectively, in the first stationary phase. For LSLI, LSHI, and HSHI cultures, EPA content increased further to 7.2, 7.1, and 5.8% DW, respectively, in the second stationary phase, while it decreased in the HSLI conditions to 5.2% DW. A similar pattern with increase from the exponential phase to the first stationary phase in all cultures and further increase to the second stationary phase in LSLI, LSHI and HSHI cultures, and decrease from the first to the second stationary phase in HSLI cultures was found for the TFA content. Corresponding values with standard deviation can be found in the Supplementary Material (Table S2). In exponential phase, TFA and EPA contents were higher in HS cultures, while after 5 days of stationary phase, levels were higher for LS cultures. EPA and TFA contents (% DW), estimated by the model, are shown in Figure S1 in the Supplementary Material.

The mathematical models, representing the EPA and the TFA content as a function of salinity (X1), irradiance (X2), growth phase (X3), and their combinations (X1X2, X1X3, X2X3) are expressed by Equations (4, 5), respectively. A positive term for the combinations in the equations indicates a synergistic effect (increasing values increase the content), whereas negative terms indicate an antagonistic effect (increasing values decrease the content; Chen et al., 2012). Coefficients in bold indicate statistical significance for the corresponding coefficients (black: *p* < 0.05 and red *p* < 0.01). For the EPA content, coefficients for growth phase, salinity, and the combination of salinity and growth phase were statistically significant whilst the other factors were not. The most significant variable with highest impact on EPA content was the growth phase with a positive estimated effect of 1.74 (*p* = 2.0*10^−13^). The combined effect of salinity and growth phase was lower (−0.48, *p* = 0.00003) and negative, while salinity had a less negative effect on EPA content (−0.19, *p* = 0.0241). The TFA content was also significantly influenced by growth phase (8.34, *p* = 1.1*10^−15^), salinity (−0.77, *p* = 0.0116), and the combination of salinity and growth phase (−1.43, *p* = 0.0002), and additionally, by the combination of salinity and irradiance (0.73, *p* = 0.0126). A high correlation (*R*^2^ = 0.9651 and 0.9802 for EPA and TFA content, respectively) of estimated values by the model and measured values indicates a good fit between the model and the experimental data (Figure S2). More details on measured and estimated values can be found in the Supplementary Material (Table S2).

(4) $$\begin{array}{l} \text{EPA}\%\;{\text{DW}}_{\text{est}}=4.61-0.19*X1-0.12*\text{X2}+1.74*X3\\ \;+0.14*\text{X1X2}-0.48*X1X3+0.02*\text{X}2\text{X}3 \end{array}$$

(5) $$\begin{array}{l} \text{TFA}\%\;{\text{DW}}_{\text{est}}=18.74-0.77*X1-0.25*\text{X2}+8.34*X3\\ \;+0.73*X1X2-1.43*X1X3-0.26*\text{X}2\text{X}3 \end{array}$$

### Batch experiment—relative fatty acid (FA) composition

**Figure 4:**
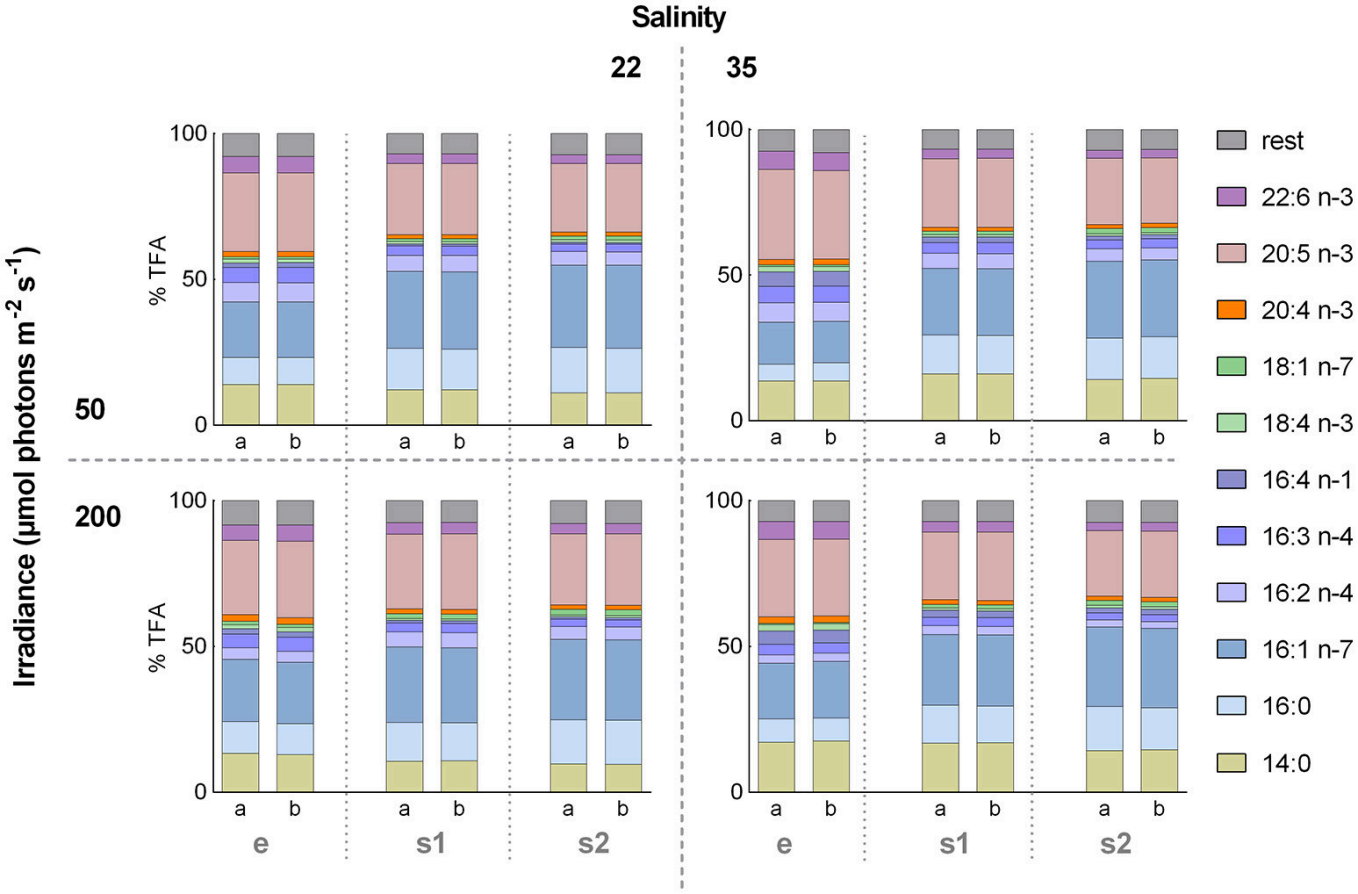
Relative fatty acid (FA) composition during factorial-design batch experiment. Effect of the 12 different treatments on the relative FA composition (% of total fatty acids [TFA]) of the diatom *Attheya septentrionalis* (each with two biological replicates, a and b). Treatments were altering combinations of the three factors salinity (22 and 35), irradiance (50 and 200 μmol photons m^−2^ s^−1^) and growth phase (exponential [e], 3 days stationary phase [s1], and 5 days stationary phase [s2]). Values are average of three measurement replicates.

**Figure 5:**
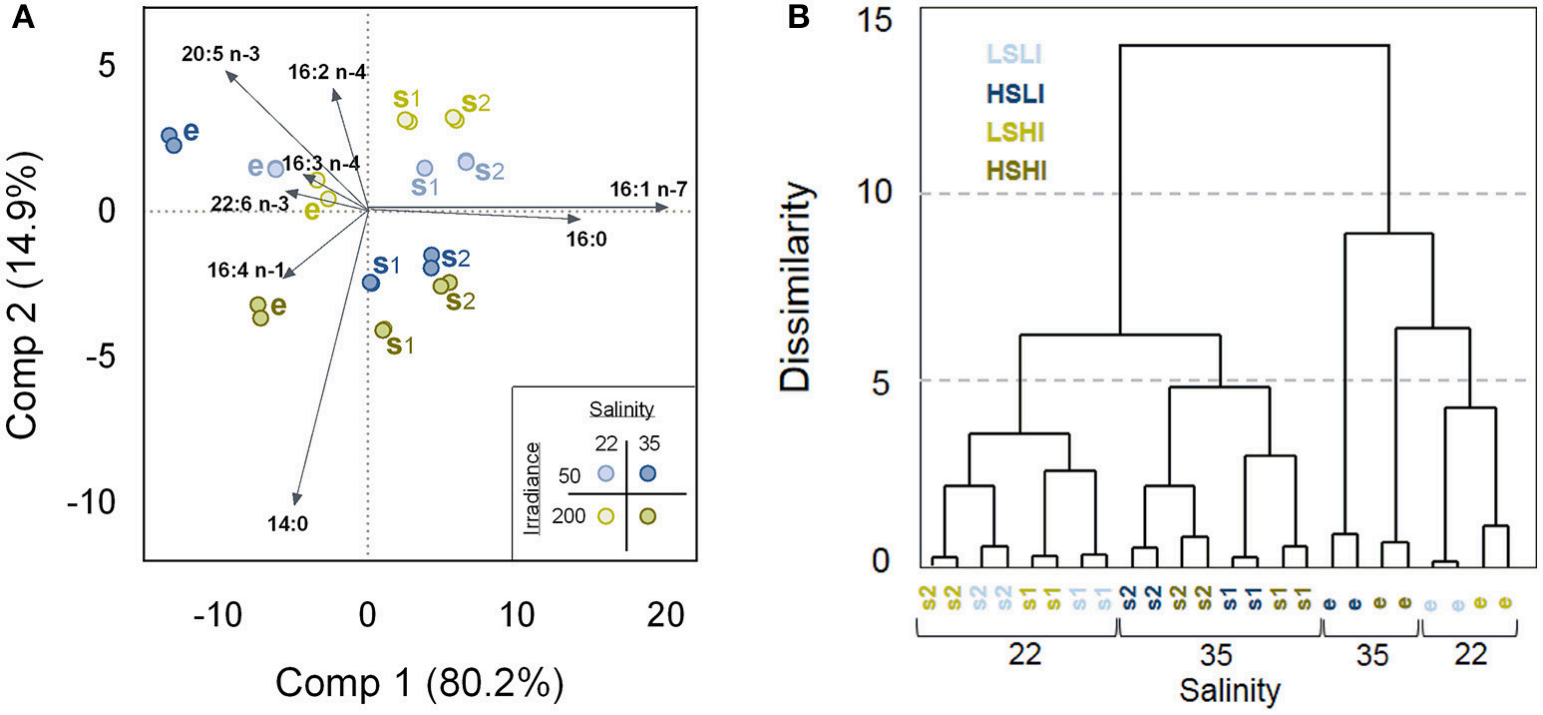
Similarities and differences in relative fatty acids (FA) composition (% of total fatty acids [TFA]) of *Attheya septentrionalis* cultures at 12 treatments during the factorial-design batch experiment (each with two biological replicates). Treatments were altering combinations of the three factors salinity (22 and 35), irradiance (50 and 200 μmol photons m^−2^ s^−1^) and growth phase (e, s1, s2). **(A)** Principal component analysis (PCA). Twenty-four objects representing the 12 treatment groups, and eight variables, representing the FAs with highest impact on the distributions. Values are average of three measurement replicates. e, exponential phase; s1, first stationary phase (Day 3); s2, second stationary phase (Day 5). **(B)** Euclidean Dendrogram showing dissimilarities between the treatment groups. LSLI, low salinity and low irradiance; HSLI, high salinity and low irradiance; LSHI, low salinity and high irradiance; HSHI, high salinity and high irradiance.

In total 36 FAs were detected in the GC for *A. septentrionalis*, from which 11 (14:0, 16:0, 16:1 n−7, 16:2 n−4, 16:3 n−4, 16:4 n−1, 18:1 n−7, 18:4 n−3, 20:4 n−3, 20:5 n−3, and 22:6 n−3) constituted more than 1% TFA (Figure 4). C16-FA were the most abundant, whereas C18-FA were present in only low amounts. In all treatments, palmitoleic acid (16:1 n−7), and EPA (20:5 n−3) were the two major FA, together accounting for between 45 and 52% TFA, followed by myristic acid (14:0) with 10–18% TFA, palmitic acid (16:0) with 6–16% TFA, and DHA (22:6 n−3) with 3–6% TFA. However, small variations in the relative FA content between the different treatment groups were apparent. These differences became more distinct by means of a principal component analysis (PCA, Figure 5A). The distribution of the 12 different treatment groups (objects) represents their similarities and differences in the relative FA composition (% TFA), and the distribution of the FAs (vectors) indicate their contribution to the grouping of the objects.

Objects were arranged along component 1 and component 2 according to the growth phases and salinities, respectively. The exponential phase objects, grouping on the left side of component 1, were clearly separated from the stationary phase objects, which clustered on the right side, and objects were shifted further to the right along component 1 with increasing nutrient starvation (stationary phases). Palmitic and palmitoleic acids were correlated positively with stationary phase samples while the PUFAs were correlated with exponential phase samples. Except for the treatment HSLI_e (high-salinity, low-irradiance, and exponential phase), treatments of the same salinity were grouped together, with LS samples on the upper region along component 2, and HS samples arranged on the lower part of component 2. Myristic acid, and to a lesser extent hexadecatetraenoic acid (16:4 n-1) were correlated positively with HS treatments, while hexadecadienoic acid (16:2 n-4) was correlated positively with LS samples. Irradiance had only small effect on the FA composition.

The Euclidean Dendrogram illustrates the distinct grouping of the treatments (Figure 5B). Exponential phase samples were separated from stationary phase samples and both groups were then further divided according to the salinity treatment. For stationary phase samples, all LS samples grouped separately from the HS samples, followed by grouping according to their growth phase (s1 and s2) and finally by irradiance. Within exponential phase samples, HSHI samples grouped between HSLI and the LS samples, and separation was followed by irradiance, where replicates of LI were separated from the HI replicates. The two biological replicates of each treatment group were very similar.

## Discussion

### Impact of culture conditions on growth

Growth rates during the pre-cultivation were strongly dependent on the salinity, followed by irradiance and the interaction of both factors. Growth rates were 43% (LI) and 72% higher (HI) at HS compared to LS, and 5% (LS) and 27% (HS) higher when grown at HI compared to LI. The negligible effect of HI on the growth rate at LS, and the much stronger effect it caused at HS conditions, reveals the combined effect of salinity and irradiance and emphasizes the importance of investigating combinatory effects of different growth factors.

These growth characteristics during the pre-cultivation also became evident when considering the growth curves during the batch experiment. The transition from exponential to stationary phase was defined by the decline of the growth curve with a concomitant decrease in the QY and was one day earlier for HS than for LS cultures. The QY reflects the photosynthetic performance of photosystem II and is used as a vitality indicator for cultures, as decreasing values are associated with stressful growth conditions (Maxwell and Johnson, 2000; Kräbs and Büchel, 2011). During exponential growth, QYs were slightly lower for LS cultures, but decreased to a lesser extent in stationary phase than they did for HS cultures. The strongest decrease in QY was observed for cultures grown at HS and HI. Hence, while the combination HSHI appeared most advantageous during nutrient replete conditions as it caused the highest growth rates in exponential phase, it was also most stressful for the cultures during nutrient starvation.

After the transition from exponential to stationary phase, cell division was assumed to have stopped due to nutrient depletion. Although all nutrients had been consumed on Day 3 in stationary phase, silicate most likely became the major limiting nutrient, as almost half of the silicate was consumed after one day in exponential phase, whereas nitrate and phosphate were both consumed to a lesser extent. When one element becomes limiting, other elements, that are more abundant, may be accumulated in the cell (Reynolds, 2006). Therefore, nitrate and phosphate, whilst not necessarily limiting, might have been taken up by the microalgae cells after silicate was depleted. In contrast to other elements that are essential for survival, diatoms rarely take up more silicon than is required for cell division (Reynolds, 2006). When silicon becomes scarce, its uptake depends on special silicic transport proteins (SITs); however, when silicon is abundant, its uptake is by diffusion (Shrestha and Hildebrand, 2015).

### EPA content during batch experiment

Within the range of experimental variables considered, three factors were identified by the model as having affected the EPA content in the present *A. septentrionalis* strain significantly; the growth phase, salinity, and the interaction of both. Growth phase had the greatest impact, with increasing nutrient starvation leading to a higher EPA content relative to DW. The effect of salinity and the combined effects of both salinity and growth phase were lower and negative. Irradiances used in this experimental set-up did not affect the EPA content significantly. The mathematical model reflected the measured EPA values very accurately, with one exception: during the experiment, the EPA content decreased slightly from first to second stationary phase under HSLI conditions, while the model predicted a further increase, similar to under the other conditions. Both the measured data and the model emphasized, that combining LS with 5 days' nutrient starvation yielded a maximum EPA content of 7.1% DW on average. This DW-based EPA content is, to our knowledge, higher than previously reported for microalgae (Sukenik et al., 1991; Lu et al., 2001; Jiang and Gao, 2004; Hu and Gao, 2006; Patil et al., 2006).

The EPA dynamics revealed the same pattern as for the TFA, suggesting that they were triggered by the same processes. Following exponential growth, the DW continued to increase between 5 and 20% from the first to the second stationary phase, although nutrients were depleted, and accordingly cell division inhibited. When cell division is hampered due to an insufficient nutrient supply, microalgae often produce carbonaceous storage compounds like carbohydrates and lipids. Many diatoms accumulate neutral storage lipids in the form of triacylglycerol (TAG), causing the lipid content to increase up to 50% of DW (Hu et al., 2008). Total FA content increased in all cultures from on average 10% in exponential phase, to 25 and 27% DW in the two stationary phases. Storage lipids consist predominantly of saturated and monounsaturated FAs, while PUFAs are generally present in polar membrane lipids (Olofsson et al., 2012). Therefore, TAG accumulation is typically accompanied by a noticeable increase of both palmitic (16:0) and palmitoleic acids (16:1 n-7), which often constitute the predominant FA in the TAG. Yet, PUFAs have also been reported to accumulate in TAG in different microalgae species (Tonon et al., 2002; Sharma et al., 2012). The relative FA compositions (% TFA) observed during the experiment revealed a slight increase of the palmitic and palmitoleic acid fractions, together with a weak decrease of PUFAs after cultures progressed from exponential to stationary phase in all conditions. However, these shifts toward palmitic and palmitoleic acids were lower than typically observed during TAG accumulation, and revealed a concurrent increase of all major FA. This might indicate that TAG accumulated in the cells during stationary phase, containing PUFAs such as EPA, alongside palmitic, and palmitoleic acids.

Another reason for the increase of the FA fraction in stationary phase might be a decrease of silica in the cells. As a result of the silicified cell walls of diatoms, silicate availability in the medium is a key factor regulating their growth, as cells can only divide when new valves can be synthesized (Martin-Jézéquel et al., 2000). Studies have shown that in silicate-limited diatom cultures, uptake is restricted to the SITs (Shrestha and Hildebrand, 2015), and silicification is reduced, resulting in thinner cell walls and a decreased silica content per cell (Martin-Jézéquel et al., 2000; Javaheri et al., 2015). Knuckey et al. (2002) found comparable results to our findings in an *A. septentrionalis* isolate from coastal waters in Tasmania, with an EPA content increasing from 1.3 to 4.2% DW from exponential to stationary phase. Concomitantly, the ash content fell sharply from 26.1 to 8.8% DW resulting in a corresponding increase of the other major organic fractions; proteins, carbohydrates, and lipids. The decreased ash content was most likely related to a diminished silica content of the DW, due to silicate limitation in the stationary phase. The same effect might have contributed to the observed increase in TFA and EPA contents relative to DW in the stationary phase in our study. The typical ash content of microalgae contributes between 5 and 12% DW, but these values are higher in silicified diatoms, between 20 and 55% DW (Nalewajko, 1966; Renaud and Parry, 1994), where much of it is attributable to the extent of silicon in the cell walls. Hildebrand et al. (2012) stated that expressing the FA content as percentage of DW might underestimate the actual amount of FA in diatoms in terms of a per cell carbon basis, when compared with other microalgae, due to their high silica content. Expressing the FA content relative to the ash-free DW might preclude such an underestimation and furthermore might give a better understanding of the FA content and dynamics during silicate-replete and silicate-depleted conditions.

Salinity also affected the TFA and EPA content, especially in combination with the growth phase. Interestingly, TFA and EPA contents were higher for HS cultures in exponential phase, but in contrast were higher for LS cultures in stationary phase. Hence, a greater increase in TFA and EPA from exponential to stationary phase occurred in the LS cultures. These observations might also be linked to differences in the FA accumulation and silica content of cells grown at different salinities, but might also be related to the changing FA composition of membrane lipids, as an adaptation to variable salt concentrations and the resultant osmotic stress, as has been reported in several studies (Chen et al., 2008; Kumari et al., 2013). Microalgae grown at higher salinities might also reveal an increased ash content, due to an increased ion concentration (Renaud and Parry, 1994).

Determining the ash and silicon contents of the cells and differentiating between polar (membrane) and neutral (storage) lipids, and their FA compositions in future experiments, might contribute to a better understanding of the reasons we observe different EPA and TFA contents relative to DW under different salinities and growth phases, and furthermore, might reveal in which lipid fraction the increased EPA levels are located.

### Relative fatty acid (FA) composition during batch experiment

Interestingly, irradiance did not significantly affect the EPA content relative to DW, although irradiances have been shown to affect photosynthetic membranes. Generally, photosynthetic membranes increase at low irradiance and are reduced at high irradiances and hence, increasing irradiance has been reported to result in a decrease in EPA and other PUFAs in different microalgae species (Adlerstein et al., 1997; Fábregas et al., 2004). However, irradiance did affect the FA profile in this study, although only to a minor degree. The relative amounts of the main FAs (% TFA) were for the most part affected by nutrient availability primarily, followed by salinity, time of nutrient starvation (days in stationary phase) and irradiance. The differences between the growth phases were mainly due to palmitic and palmitoleic acids and therefore might be related to an accumulation of TAG in stationary phase. The effects of the growth conditions (salinity and irradiance) were less distinct and are more difficult to explain, but might be related to reconstructions of cellular membranes as an adaptation to the cultivation conditions.

### Potential for microalgae-based technologies

The low salinity of 22 was more effective in increasing EPA content in the stationary phase in the prevalent *A. septentrionalis* strain, but at the same time, it considerably decreased growth rates compared to a HS of 35. In future large-scale cultivations, EPA productivity would be dependent on both the growth rates and the EPA content in the cells. Calculating the EPA productivities from exponential phase until Day 5 of the stationary phase revealed 0.97 mg L^−1^ d^−1^ for LS cultures and 0.72 mg L^−1^ d^−1^ for HS cultures. Thus, under the prevailing conditions, higher productivities were obtained for the LS cultures, although these productivities are much lower than those seen in commercial production due to the much lower biomass concentrations used in this experimental setup. Whether our results can be successfully repeated in up-scaled systems needs to be evaluated further. Higher nutrient concentrations would be necessary in order to achieve higher cell densities and productivities before cultures reach the stationary phase. Furthermore, other growth conditions such as irradiance, temperature and pH might change considerably when moving from small-scale to large-scale systems, and can thereby affect the EPA content of the cells. The strain used in the current study was isolated from an Arctic habitat and adapted to low temperatures, and therefore temperatures in the experiment were maintained at 10°C. Several studies have shown that low temperatures can increase the PUFA content to maintain membrane fluidity (Boelen et al., 2013). EPA values in the present experiments were higher than values recorded for the *A. septentrionalis* strain by Knuckey et al. (2002) grown at 20°C. However, at the same time, growth rates of their strain were twice as high as the ones observed in this study. Hence, changing temperatures could additionally affect both the EPA content and growth rates. This should also be evaluated with further work.

Knuckey et al. (2002) suggested *A. septentrionalis* to be an excellent feed species for juvenile bivalve molluscs and other filter feeders. Its cell size is within the range that is suitable for ingestion by filter feeders and its protein level (32% DW) remained stable from exponential to stationary phase, while carbohydrate and lipid fractions increased. As shown in our study, EPA contents can be further increased in stationary phase, by changing growth conditions. This could make this diatom strain a promising EPA source for the North Atlantic fish aquaculture industry or for other application areas, such as the health and food sectors.

## Conclusion

The effect of growth phase, salinity and irradiance, and their interactions on the EPA content in an Arctic *A. septentrionalis* strain was investigated by means of a factorial design experiment. The highest EPA values of 7.1% DW were achieved at a salinity of 22 and Day 5 of the stationary phase. However, at the same time, growth rates during exponential phase were reduced considerably at low salinities. Mathematical models revealed interactive effects of salinity and irradiance on growth and of salinity and growth phase on the EPA content, emphasizing the importance of investigating the additive effects of different growth factors.

## Author contributions

PS, SP, SM, and SE: designed research; PS: performed research; PS and SM: analyzed data; PS: wrote the paper; SE and SM: revised the paper. All authors read and approved the final manuscript.

### Conflict of interest statement

The authors declare that the research was conducted in the absence of any commercial or financial relationships that could be construed as a potential conflict of interest.
