# Supplementary material for: Enhancing EPA Content in an Arctic Diatom: A Factorial Design Study to Evaluate Interactive Effects of Growth Factors
Source: Front Plant Sci. 2018 Apr 17;9:491. doi: 10.3389/fpls.2018.00491 (PMC5932356; doi:10.3389/fpls.2018.00491)
Supplement: Supplementary file 1 [file Table1.PDF]

Supplementary Material

Enhancing EPA content in an Arctic diatom: A factorial design study to evaluate interactive effects of growth factors

Pia Steinrücken*, Svein Are Mjøs, Siv Kristin Prestegard, Svein Rune Erga

*** Correspondence:** Pia Steinrücken: pia.steinrucken@uib.no

2 Supplementary Tables

Table S1. Growth rates (µ) during pre-cultivation. Growth rates of the last seven repeated dilutions and estimated growth rates by the mathematical model of the diatom *Attheya septentrionalis* grown at four different cultivation conditions. LSLI: Low salinity and low irradiance (µmol photons m^{-2} s^{-1}), HSLI: High salinity and low irradiance, LSHI: Low salinity and high irradiance, HSHI: High salinity and high irradiance.

	Factors			Measurements							Model		
No	Salinity X1	Irradiance X2	Condition	µ (d^{-1}) 1	2	3	4	5	6	7	Coded levels X1	X2	µ (d^{-1}) estimated
1	22	50	LSLI	0.55	0.62	0.60	0.56	0.46	0.56	0.45	-1	-1	0.53
2	35	50	HSLI	0.68	0.80	0.78	0.76	0.75	0.86	0.79	1	-1	0.77
3	22	200	LSHI	0.66	0.78	0.54	0.53	0.45	0.47	0.57	-1	1	0.57
4	35	200	HSHI	0.90	1.00	1.09	0.93	0.92	1.08	0.96	1	1	0.98
