# Supplementary material for: Enhancing EPA Content in an Arctic Diatom: A Factorial Design Study to Evaluate Interactive Effects of Growth Factors
Source: Front Plant Sci. 2018 Apr 17;9:491. doi: 10.3389/fpls.2018.00491 (PMC5932356; doi:10.3389/fpls.2018.00491)
Supplement: Supplementary file 2 [file Table2.pdf]

Supplementary Material

Enhancing EPA content in an Arctic diatom: A factorial design study to evaluate interactive effects of growth factors

Pia Steinrücken*, Svein Are Mjøs, Siv Kristin Prestegard, Svein Rune Erga

*** Correspondence:** Pia Steinrücken: pia.steinrucken@uib.no

2 Supplementary Tables

Table S2. Concept and results of the factorial-design batch experiment. LSLI: low salinity and low irradiance (μmol photons m^{-2} s^{-1}), HSLI: high salinity and low irradiance, LSHI: low salinity and high irradiance, HSHI: High salinity and high irradiance, e: exponential phase, s1: first stationary phase (Day 3), s2: second stationary phase (Day 5). DW: dry weight, TFA: total fatty acids, EPA: eicosapentaenoic acid, Av: average of three measurement replicates, SD: standard deviation of three measurement replicates.

	Factors				Measurements									Model				
No	Salinity X1	Irradiance X2	Growth phase X3	Treatment group	Quantum Yield	DW		TFA % DW		EPA % DW		EPA %TFA		Coded levels			TFA% DW estimated	EPA% DW estimated
						Av	SD	Av	SD	Av	SD	Av	SD	X1	X2	X3		
1a	22	50	exponential	LSLI_e	0.64	0.09	0.01	10.6	1.9	2.9	0.5	27.0	0.1	-1	-1	-1	10.46	2.85
1b					0.64	0.09	0.01	9.9	1.6	2.7	0.4	27.0	0.4					
2a	22	50	stationary 1	LSLI_s1	0.58	0.23	0.01	26.7	1.2	6.5	0.3	24.4	0.2	-1	-1	0.74	27.93	6.69
2b					0.57	0.21	0.01	29.5	2.2	7.2	0.6	24.4	0.2					
3a	22	50	stationary 2	LSLI_s2	0.56	0.27	0.01	29.8	1.6	7.0	0.4	23.5	0.1	-1	-1	1	30.54	7.27
3b					0.56	0.26	0.01	31.4	1.7	7.4	0.4	23.5	0.2					
4a	35	50	exponential	HSLI_e	0.69	0.07	0.00	11.0	0.5	3.4	0.2	30.8	0.6	1	-1	-1	10.32	3.15
4b					0.69	0.08	0.00	9.7	0.8	2.9	0.3	30.3	0.7					
5a	35	50	stationary 1	HSLI_s1	0.56	0.19	0.00	24.5	1.5	5.8	0.4	23.5	0.0	1	-1	0.74	22.79	5.32
5b					0.57	0.18	0.01	24.1	1.6	5.7	0.3	23.7	0.4					
6a	35	50	stationary 2	HSLI_s2	0.53	0.21	0.00	24.3	1.0	5.5	0.2	22.7	0.1	1	-1	1	24.65	5.65
6b					0.53	0.22	0.01	21.9	1.0	4.9	0.3	22.5	0.1					
7a	22	200	exponential	LSHI_e	0.64	0.08	0.02	9.8	2.5	2.5	0.7	25.4	0.4	-1	1	-1	9.02	2.30
7b					0.64	0.08	0.00	9.0	0.9	2.4	0.2	26.2	0.1					
8a	22	200	stationary 1	LSHI_s1	0.49	0.21	0.01	24.5	1.5	6.1	0.6	25.7	0.3	-1	1	0.74	25.57	6.20
8b					0.49	0.22	0.02	24.1	1.6	5.5	0.7	25.9	0.2					
9a	22	200	stationary 2	LSHI_s2	0.53	0.22	0.01	30.0	3.5	7.3	0.8	24.4	0.2	-1	1	1	28.04	6.78
6b					0.52	0.23	0.01	27.8	2.7	6.8	0.7	24.5	0.1					
10a	35	200	exponential	HSHI_e	0.68	0.08	0.00	11.0	0.7	2.9	0.2	26.6	0.4	1	1	-1	11.81	3.15
10b					0.68	0.07	0.01	12.1	2.0	3.2	0.5	26.4	0.5					
11a	35	200	stationary 1	HSHI_s1	0.46	0.18	0.00	23.8	0.6	5.5	0.2	23.2	0.0	1	1	0.74	23.37	5.37
11b					0.43	0.18	0.01	23.1	1.6	5.4	0.4	23.4	0.2					
12a	35	200	stationary 2	HSHI_s2	0.47	0.20	0.01	25.9	1.7	5.8	0.4	22.4	0.3	1	1	1	25.09	5.70
12b					0.44	0.21	0.01	24.6	1.0	5.8	0.2	22.7	0.1					

frontiers
