# Supplementary material for: Enhancing EPA Content in an Arctic Diatom: A Factorial Design Study to Evaluate Interactive Effects of Growth Factors
Source: Front Plant Sci. 2018 Apr 17;9:491. doi: 10.3389/fpls.2018.00491 (PMC5932356; doi:10.3389/fpls.2018.00491)
Supplement: Supplementary file 3 [file Image1.pdf]

Supplementary Material

Enhancing EPA content in an Arctic diatom: A factorial design study to evaluate interactive effects of growth factors

Pia Steinrücken*, Svein Are Mjøs, Siv Kristin Prestegard, Svein Rune Erga

*** Correspondence:** Pia Steinrücken: pia.steinrucken@uib.no

1 Supplementary Figures

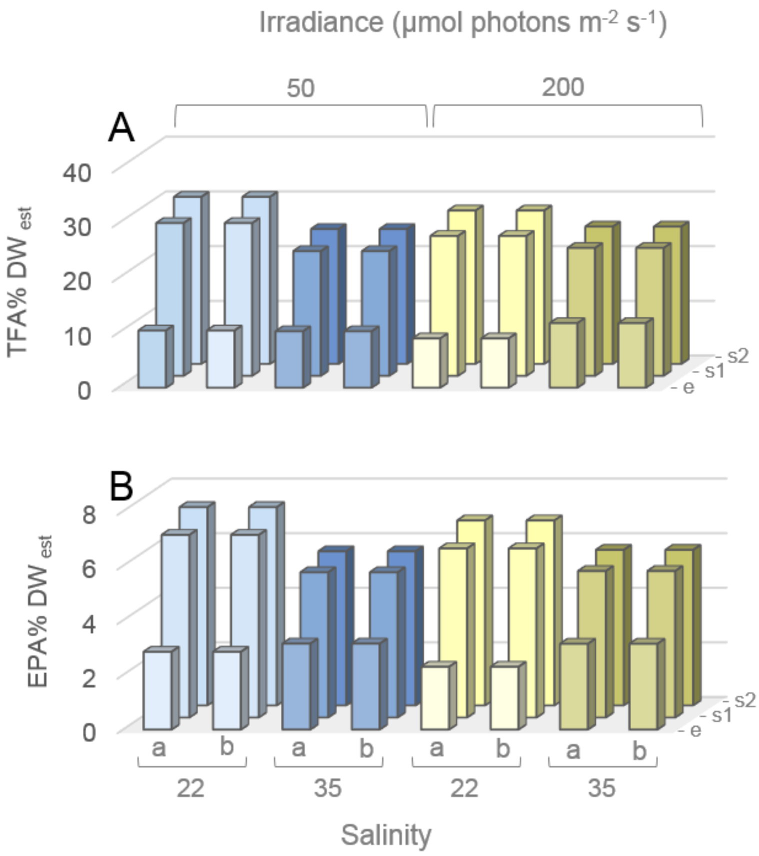

Fig. S1. Model-estimated total fatty acid (TFA) (**A**) and EPA contents (**B**) relative to the dry weight (DW) of *Attheya septentrionalis*, at twelve different treatments (each with two biological replicates). e: exponential phase, s1: first stationary phase (Day3), s2: second stationary phase (Day 5).
