# Supplementary material for: Enhancing EPA Content in an Arctic Diatom: A Factorial Design Study to Evaluate Interactive Effects of Growth Factors
Source: Front Plant Sci. 2018 Apr 17;9:491. doi: 10.3389/fpls.2018.00491 (PMC5932356; doi:10.3389/fpls.2018.00491)
Supplement: Supplementary file 4 [file Image2.pdf]

frontiers

Supplementary Material

Enhancing EPA content in an Arctic diatom: A factorial design study to evaluate interactive effects of growth factors

Pia Steinrücken*, Svein Are Mjøs, Siv Kristin Prestegard, Svein Rune Erga

*** Correspondence:** Pia Steinrücken: pia.steinrucken@uib.no

1 Supplementary Figures

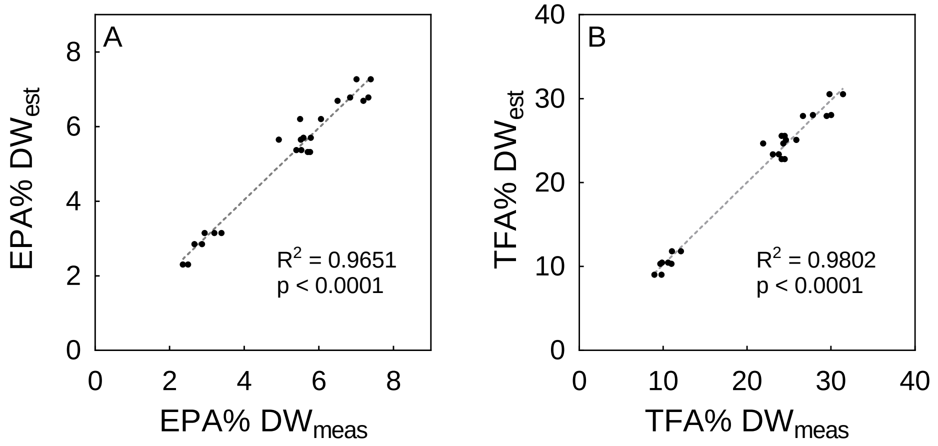

Fig. S2. Measured EPA (A) and total fatty acid (TFA) content (B) during the factorial-design experiment, plotted against the estimates by the mathematical model with linear regression and R^2 value with high significance. **Meas**: measured values, **est**: values estimated by the model.

frontiers
